# Cross-cultural adaptation and psychometric validation of the Arabic Leicester Cough Questionnaire in patients with chronic cough: A cross-sectional study

**DOI:** 10.1097/MD.0000000000050891

**Published:** 2026-10-02

**Authors:** Saeed M. Alghamdi, Ahmed H. Alasimi, Maryam M. Almulhem, Saja A. Almulhim, Abdulelah M. Aldhahir, Jaber S. Alqahtani, Rayan A. Siraj, Surinder S. Birring

**Affiliations:** a Respiratory Care Program, Clinical Technology Department, Faculty of Applied Medical Sciences, Umm Al-Qura University, Makkah, Saudi Arabia; b National Heart and Lung Institute, Imperial College London, London, UK; c Respiratory Therapy Department, College of Applied Medical Sciences, King Faisal University, Alahsa, Saudi Arabia; d Respiratory Therapy Program Department, College of Applied Medical Sciences, King Saud bin Abdulaziz University for Health Sciences (KSAU-HS), Alahsa, Saudi Arabia; e Respiratory Therapy Program, Department of Nursing, College of Nursing and Health Sciences, Jazan University, Jazan, Saudi Arabia; f Department of Respiratory Care, Prince Sultan Military College of Health Sciences, Dhahran, Saudi Arabia; g Research and Innovation Institute, Ministry of Defense Health Services, Riyadh, Saudi Arabia; h Centre for Human and Applied Physiological Sciences, Faculty of Life Sciences & Medicine, King’s College London, London, United Kingdom.

**Keywords:** Arabic version, chronic cough, cross-cultural adaptation, LCQ, Leicester Cough Questionnaire, psychometric validation, reliability, respiratory care, Saudi Arabia

## Abstract

Chronic cough significantly impacts patients’ quality of life, yet validated Arabic-language tools for its assessment are lacking. This study aimed to translate, culturally adapt, and validate the Arabic version of the Leicester Cough Questionnaire (LCQ-A) among Arabic-speaking patients. This cross-sectional study involved the translation, cross-cultural adaptation, and psychometric validation of the LCQ among Arabic-speaking patients with chronic cough. Participants completed the LCQ-A and the COPD Assessment Test (CAT). Internal consistency was assessed using Cronbach’s α, test–retest reliability using intraclass correlation coefficients, and convergent validity via Spearman’s correlation with the CAT cough item. A total of 56 adult patients with chronic cough were recruited with a mean age of 28.5 ± 4.8 years. Clinical causes or conditions associated with chronic cough included postinfection cough (26.8%), sinusitis (17.9%), asthma (14.3%), and an unknown cause (41.1%). The mean LCQ-A total score was 16.9 ± 3.7, with subdomain scores of 5.3 ± 1.3 (physical), 5.9 ± 1.2 (psychological), and 5.8 ± 1.5 (social). Cronbach’s α demonstrated excellent internal consistency across domains (physical, 0.870; psychological, 0.882; social, 0.925; total, 0.952). Test–retest reliability of the LCQ-A in participants with no change in cough symptoms was excellent, with intraclass correlation coefficient values ranging from 0.859 to 0.944 for single measures and 0.924 to 0.971 for average measures (all *P* < .001). Strong interdomain correlations were observed (ρ = 0.877–0.957, *P* < .001), indicating high internal coherence. However, LCQ-A scores did not significantly correlate with the CAT cough item (ρ = −0.030 to 0.057, *P* > .05). The LCQ-A demonstrates excellent reliability and strong internal consistency, confirming its suitability for assessing cough-related quality of life in Arabic-speaking populations. While convergent validity with the CAT cough item was not observed, the LCQ-A provides a culturally adapted and psychometrically robust instrument for clinical and research use.

**Current knowledge**

Chronic cough is a common respiratory symptom that significantly impairs quality of life. The Leicester Cough Questionnaire (LCQ) is a widely used, validated tool in English and several other languages to assess cough-specific quality of life. However, no validated Arabic version of the LCQ has been available for use in clinical or research settings.

**What this paper contributes to our knowledge**

The Arabic version of the LCQ (LCQ-A) demonstrated excellent internal consistency and strong test–retest reliability among patients with chronic cough. Although it did not significantly correlate with the cough item of the COPD Assessment Test, the LCQ-A provides a reliable and culturally adapted instrument for assessing cough-related quality of life in Arabic-speaking populations.

## 1. Introduction

Cough is one of the most frequent respiratory symptoms prompting patients to seek medical attention in both respiratory and primary care settings.^[1]^ According to the European Respiratory Society guidelines, a cough persisting for more than 8 weeks is defined as a chronic cough.^[2]^ Chronic cough is often associated with underlying conditions such as chronic obstructive pulmonary disease (COPD), asthma, and gastroesophageal reflux disease, although chronic cough may persist even after optimal treatment of the underlying cause.^[3–5]^ Globally, chronic cough affects approximately 10% of the population, with a higher prevalence observed among females and older adults.^[6]^ Similarly, a study conducted at a chest clinic in Riyadh, Saudi Arabia, reported a prevalence of 10%.^[7]^ Chronic cough significantly impairs patients’ quality of life and is frequently associated with psychological distress.^[8–10]^ Therefore, evaluating its impact using multidimensional outcome measures is essential to capture the full burden of the condition.

Patient-reported outcome measures (PROMs) are widely utilized to obtain a comprehensive understanding of the impact of chronic cough on patients’ quality of life.^[11,12]^ The Leicester Cough Questionnaire (LCQ) is an English-language PROM specifically developed to assess the impact of chronic cough over the preceding 2 weeks across 3 domains: psychological, social, and physical.^[13]^ The LCQ is concise, typically requiring less than 5 minutes to complete, which makes it practical for use in both clinical and research settings.^[13]^ Previous studies have demonstrated the validity, reliability, and responsiveness of the LCQ in measuring the burden of chronic cough in adults.^[13–15]^ Furthermore, the LCQ has been translated and validated for use in multiple languages,^[16–19]^ and its validity has also been established across several respiratory conditions including COPD and cystic fibrosis.^[20,21]^

Arabic is spoken by more than 250 million people across the Middle East and North Africa; however, there is a lack of validated Arabic-language tools for assessing chronic cough. Translating and validating the LCQ into Arabic will address this gap, enabling clinicians and researchers to assess the impact of chronic cough in a reliable and culturally appropriate way. Accordingly, the present study aimed to translate the LCQ into Arabic (LCQ-A) and to assess its validity and reliability.

## 2. Methods

### 2.1. Study design and setting

This cross-sectional study was conducted at the Security Forces Hospital (SFH) in Makkah, Saudi Arabia, from February 2023 to May 2025. It involved a cross-cultural adaptation of the LCQ to accurately capture cultural norms unique to Arabic-speaking patients with chronic cough. The reporting of the study follows the Strengthening the Reporting of Observational studies in Epidemiology guidelines.^[22]^

### 2.2. Translation and cultural adaptation

The LCQ was translated into Arabic following a standardized forward–backward translation procedure. Two independent bilingual translators first produced forward translations from English to Arabic, which were then reconciled into a single preliminary version by an expert panel comprising respiratory physicians, registered respiratory therapists, and linguists. A backward translation into English was subsequently performed by a native English speaker fluent in Arabic with a medical background. This version was compared with the original LCQ to ensure semantic, conceptual, and cultural equivalence.

Pre-testing of the Arabic version (LCQ-A) was conducted with a small group of patients to evaluate clarity, comprehensibility, and cultural appropriateness. Minor revisions were made based on patient feedback, after which the translators confirmed that the final wording accurately conveyed the intended meaning of the original questionnaire, ensuring overall equivalence.

### 2.3. Participants

Adult patients with chronic cough (lasting more than 8 weeks) were recruited consecutively from the pulmonary clinics at SFH. Eligible participants were aged ≥18 years, had chronic cough diagnosed by a pulmonary consultant, demonstrated bilingual proficiency in Arabic and English, and were able to provide informed consent. Patients were excluded if they were healthy smokers or had an active chest infection, psychiatric illness, cognitive impairment, or any condition preventing completion of self-administered questionnaires. The sample size was determined in accordance with recommendations from previous validation studies and confirmed in consultation with the original developer of the LCQ (Prof Birring), with guidelines suggesting a minimum of 5 to 10 participants per questionnaire item.^[13,17–19,21,23]^ Participants completed the LCQ-A along with comparator questionnaires during their initial clinic visit. To assess test–retest reliability, a convenience sample of 18 participants from the original cohort of 56 returned to the clinic and completed the LCQ-A again 2 weeks after the initial assessment. Participants were included in the test–retest analysis if their cough symptoms remained unchanged during the 2-week interval.

### 2.4. Evaluation of the validity and reliability of the Arabic LCQ

The validity and reliability of the LCQ-A were evaluated through established psychometric methods. Convergent validity was assessed by correlating the domain scores of the LCQ-A with the cough-specific item (Item 2) of the COPD Assessment Test (CAT) using Spearman’s rank correlation coefficient. Internal consistency was determined using Cronbach’s α, with values ≥0.70 considered acceptable, and test–retest reliability was evaluated using the intraclass correlation coefficient (ICC) to examine the stability of the LCQ-A over time.

### 2.5. Assessment of COPD burden

In addition to the LCQ-A, validated instruments were administered to evaluate convergent validity. Specifically, the CAT was used to assess symptom burden in patients with chronic respiratory conditions, including chronic cough. The CAT is a widely used PROM designed to quantify the overall impact of COPD on a patient’s health status. It comprises 8 items, each addressing a different aspect of health impairment: cough, sputum production, chest tightness, breathlessness, limitations in daily activities, confidence in leaving home, sleep quality, and energy levels. Each item is scored on a 6-point Likert scale ranging from 0 (no impairment) to 5 (severe impairment), resulting in a total score from 0 to 40, with higher scores indicating a greater disease burden. The CAT is brief, easy to complete (typically under 5 minutes), and has been validated in multiple languages, including Arabic.^[24]^

### 2.6. Ethical considerations

Ethical approval was obtained from the Institutional Review Board of the SFH in Makkah in February 2023 (ID: 0559-070223). All participants provided written informed consent prior to participation. The study adhered to the principles of the Declaration of Helsinki.

### 2.7. Statistical analysis

Data were analyzed using IBM SPSS Statistics for Windows (Version 29.0; IBM Corp.). Descriptive statistics were used to summarize participant demographics and questionnaire scores, with continuous variables presented as means ± standard deviations and categorical variables as frequencies and percentages. Normality of continuous variables was assessed using the Shapiro–Wilk test. Independent-samples *t* tests and one-way analysis of variance were used for group comparisons, while Spearman’s rank-order correlation (ρ) evaluated convergent validity between the LCQ-A and related measures, including the CAT. Internal consistency was assessed using Cronbach’s α, and test–retest reliability was determined via the ICC based on a two-way mixed-effects model. A *P* value < .05 was considered statistically significant.

## 3. Results

### 3.1. Demographic and clinical characteristics of the study participants

The study included 56 participants with a mean age of 28.5 ± 4.84 years. The majority were male (60.7%). Regarding the causes associated with chronic cough, 41.1% had no known condition, while 26.8% reported postinfection chronic cough, 17.9% had sinusitis, and 14.3% had asthma. Only 12.5% had undergone lung function testing in the past 6 months. In terms of medication use, 44.7% were not taking any medications, whereas 25.0% used antibiotics, 16.1% used expectorants, and 7.1% each used bronchodilators and inhaled antihistamines.

The LCQ-A total score had a mean of 16.9 ± 3.7, with further breakdowns across the 3 subdomains. The physical domain had a mean score of 5.3 ± 1.3, while the psychological domain had a mean of 5.9 ± 1.2. The social domain had a mean score of 5.8 ± 1.5. A detailed characterization of the study participants is presented in Table 1.

**Table 1:** Demographic and clinical characteristics of the study participants (n = 56).

Variable	Mean ± SD/n (%)
Age (yr)	28.5 ± 4.84
Gender	
Male	34 (60.7)
Female	22 (39.3)
Causes of chronic cough	
Asthma	8 (14.3)
Sinusitis	10 (17.9)
Postinfection chronic cough	15 (26.8)
Unknown	23 (41.1)
Lung function test in past 6 mo	
Yes	7 (12.5)
No	49 (87.5)
Current medications	
Bronchodilators	4 (7.1)
Inhaled antihistamines	4 (7.1)
Expectorants	9 (16.1)
Antibiotics	14 (25.0)
None	25 (44.7)
Leicester Cough Questionnaire score	16.9 ± 3.7
Physical domain	5.3 ± 1.3
Psychological domain	5.9 ± 1.2
Social domain	5.8 ± 1.5
CAT (cough item) score	3.3 ± 1.1

CAT = COPD Assessment Test, n = number, SD = standard deviation.

### 3.2. Validity of the Arabic version of the LCQ

Spearman’s rank correlation coefficient was used to evaluate the association between the LCQ-A domains and the total LCQ-A score. The results demonstrated strong and statistically significant positive correlations between the total LCQ-A score and its 3 subdomains: physical (ρ = 0.957, *P* < .001), psychological (ρ = 0.942, *P* < .001), and social (ρ = 0.953, *P* < .001), indicating a high level of interrelation among these components. Additionally, significant positive correlations were found between the physical and psychological domains (ρ = 0.877, *P* < .001), physical and social domains (ρ = 0.883, *P* < .001), and psychological and social domains (ρ = 0.878, *P* < .001). However, no significant correlations were found between any of the LCQ-A domains and the total LCQ-A score with the CAT cough item score (total LCQ-A score: ρ = −0.030, *P* = .828; physical: ρ = 0.057, *P* = .675; psychological: ρ = −0.053, *P* = .697; social: ρ = 0.050, *P* = .717). These findings suggest that while the LCQ-A demonstrates strong interdomain correlations, it does not show a significant correlation with the CAT cough item. Data are presented in Table 2.

**Table 2:** Spearman’s correlation between LCQ domains and CAT cough item.

	Physical	Psychological	Social	Total LCQ	CAT cough item
Physical		0.877 (<.001)	0.883 (<.001)	0.957 (<.001)	**0.057 (.675**)
Psychological	0.877 (<.001)		0.878 (<.001)	0.942 (<.001)	**−0.053 (.697**)
Social	0.883 (<.001)	0.878 (<.001)		0.953 (<.001)	**0.050 (.717**)
Total LCQ	0.957 (<.001)	0.942 (<.001)	0.953 (<.001)		**−0.030 (.828**)
CAT cough item	**0.057 (.675**)	**−0.053 (.697**)	**0.050 (.717**)	**−0.030 (.828**)	

Spearman’s rho (ρ) (*P* value).

Statistically significant values are shown in bold.

CAT = COPD Assessment Test, LCQ = Leicester Cough Questionnaire.

### 3.3. Reliability of the Arabic version of the LCQ

The internal consistency of the LCQ-A was evaluated using Cronbach’s α across its 3 domains: physical, psychological, and social. The findings demonstrated good to excellent reliability for all domains, confirming that the translated version maintains a high level of internal consistency. Specifically, the physical domain (8 items) showed good internal consistency (α = 0.870), reflecting strong inter-item correlations. Similarly, the psychological domain (7 items) exhibited good reliability (α = 0.882). The social domain (4 items) displayed excellent internal consistency (α = 0.925), highlighting a robust interrelationship among its items. In addition, the overall internal consistency of the total LCQ-A (19 items) was found to be excellent, with a Cronbach’s α of 0.952, further supporting the reliability of the Arabic version of the questionnaire. These Cronbach’s α values for each domain and the total LCQ-A are presented in Table 3.

**Table 3:** Reliability of LCQ domains.

LCQ	Number of items	Cronbach’s α	Reliability level
Physical	8	0.870	Good
Psychological	7	0.882	Good
Social	4	0.925	Excellent
Total LCQ	19	0.952	Excellent

### 3.4. Test–retest reliability

Test–retest reliability was assessed in 18 participants from the original cohort whose cough symptoms remained unchanged during the interval using the ICC through a two-way mixed-effects model for absolute agreement. The results demonstrated excellent stability over time. The single-measure ICC values were 0.910 for the physical domain, 0.859 for the psychological domain, 0.921 for the social domain, and 0.944 for the total score. Corresponding average-measure ICC values were 0.953 (physical), 0.924 (psychological), 0.959 (social), and 0.971 (total score), all with *P* values < .001. The 95% confidence intervals for the ICC estimates further corroborated the reliability, ranging from 0.771 to 0.983. These results confirm that the LCQ-A maintains robust internal consistency and stability over time, affirming its reliability as a tool for assessing cough-related quality of life in Arabic-speaking populations. These findings are summarized in Table 4.

**Table 4:** Test–retest reliability of LCQ-A.

Domain	ICC (single measures)	95% CI (lower–upper)	ICC (average measures)	95% CI (lower–upper)	*P* value
Physical	0.910	0.852–0.946	0.953	0.920–0.972	<.001
Psychological	0.859	0.771–0.915	0.924	0.871–0.956	<.001
Social	0.921	0.870–0.953	0.959	0.930–0.976	<.001
Total LCQ	0.944	0.906–0.966	0.971	0.951–0.983	<.001

CI = confidence interval, ICC = intraclass correlation coefficient, LCQ = Leicester Cough Questionnaire.

## 4. Discussion

This study aimed to evaluate the validity, internal consistency, and test–retest reliability of the LCQ-A among Arabic-speaking individuals with persistent cough. The validity of this questionnaire was supported by a strong correlation between LCQ-A domains and the total score, with Spearman’s rho coefficients exceeding 0.87 in all cases (*P* < .001). This indicates that all items are relevant to the overall construct of cough-related quality of life in this cultural context. The internal consistency of the LCQ-A was robust across all domains, falling within the range considered good to excellent, with Cronbach’s α ranging from 0.87 to 0.93, and for the overall score, α = 0.95. This internal consistency indicates that the translated items reliably measure the intended constructs. Test–retest reliability was strong, with ICCs above 0.85 in each domain and up to 0.94 for the total score. These results support the LCQ-A as a valid and reliable method for assessing chronic cough in Arabic-speaking populations.

Our findings are aligned with previous validation studies in other languages. The psychometric indices observed in this study fall within the same range as those reported for the original English version and other translated versions of the LCQ.^[13,16,18,19,25]^ For example, the original English LCQ reported a total score Cronbach’s α of approximately 0.92 and an ICC around 0.96, values very close to those we found.^[13]^ Similarly, validation studies in languages such as Spanish, Chinese, Korean, and Dutch, which applied the LCQ in different respiratory diseases, have all documented Cronbach’s α values in the high 0.90s, with strong test–retest stability (ICCs, 0.75–0.90 or above).^[16,18,19,25]^ Overall, the consistency of LCQ performance across languages reaffirms the cross-cultural validity of the LCQ framework for measuring the impact of chronic cough on quality of life (Table 5).

**Table 5:** Reliability, validity, and responsiveness of the LCQ across different language versions.

Language	Cronbach’s α (total)	ICC (test–retest)	Convergent validity	Responsiveness	Notes
English	0.92	0.89	Strong correlation with cough VAS and SGRQ	Yes	Original version; widely validated
Spanish	0.93	0.86	Correlation with SGRQ (*r* = −0.74)	Yes	Validated in CF bronchiectasis patients
Persian	0.974	0.983	Correlation with SF-36; RMSEA = 0.09	Yes	Validated in pulmonary sarcoidosis patients
German	0.89	Not reported	Moderate correlation with HRQoL tools	Not reported	Validated in sarcoidosis patients
Italian	Not reported	Not reported	Validated for chronic cough impact	Not reported	Recent validation study
Korean	0.85	0.88	Correlation with cough severity scales	Yes	Validated in chronic cough patients
Dutch	0.90	0.87	Correlation with cough-specific QoL measures	Yes	Validated in chronic cough patients
Chinese	0.91	0.89	Correlation with cough frequency and severity	Yes	Validated in chronic cough patients
Arabic	0.952	0.944	Weak correlation with CAT cough item	Not tested	Validity in chronic cough

CAT = COPD Assessment Test, CF = cystic fibrosis, HRQoL = health-related quality of life, ICC = intraclass correlation coefficient, QoL = quality of life, RMSEA = root mean square error of approximation, SF-36 = 36-Item Short Form Health Survey, SGRQ = St George’s Respiratory Questionnaire, VAS = visual analog scale.

In terms of construct validity, this study found strong interdomain correlations within the LCQ-A. Similar approaches have been used in other validation studies of the LCQ, supporting the interpretation that this instrument reflects the intended construct across cultures.^[17]^ On the other hand, our analysis also revealed a lack of significant correlation between LCQ-A scores and the CAT cough item score. This finding contrasts with the Swedish validation study of the LCQ, which reported a strong correlation with the CAT.^[23]^ This difference can be attributed to the distinct design and purpose of the 2 instruments. The LCQ is a cough-specific quality-of-life tool that captures physical, psychological, and social impacts across 19 items. In contrast, the CAT is a specific tool for COPD, containing only a single item related to cough among its 8 questions.^[26]^ A single-item measure of cough frequency/severity in the CAT is limited in scope compared with the multidimensional assessment provided by the LCQ. In addition, our study population included individuals with chronic cough from diverse causes and was not limited to patients with COPD. Therefore, the absence of a strong correlation between the LCQ-A and the CAT’s cough item likely reflects the fact that these instruments capture different aspects of a patient’s experience.

The quality of life in chronic cough is primarily shaped by how patients perceive and cope with their symptoms. While tools such as 24-hour cough sound monitoring exist, they are not yet validated or widely available.^[27]^ Patients often find the social and psychological impact of coughing more troubling than the physical burden.^[28]^ For this reason, the LCQ is currently the most suitable measure, as it reflects what matters most to patients and fills the gap left by the lack of reliable objective alternatives.

We acknowledge some potential limitations of this study. The use of a single “cough” item from the CAT rather than a dedicated cough severity scale for the validation procedure is one of the limitations of the study. While the CAT cough item provides some symptom context, it is less comprehensive than instruments such as a cough visual analog scale or the full cough severity index. In addition, responsiveness remains to be tested in future studies. The current cross-sectional design did not include follow-up data, so this property remains untested for the LCQ-A. Further studies are needed to determine the responsiveness of the LCQ-A and its ability to detect clinically meaningful changes over time. Another limitation is that our sample might not have captured all etiologies of chronic cough. Thus, further research in more diverse or condition-specific Arabic-speaking populations could help generalize the findings of the LCQ-A.

The strong psychometric performance of the LCQ-A in this study highlights the value of having a reliable Arabic-language instrument for assessing chronic cough. Chronic respiratory symptoms, including persistent cough, are prevalent in Middle Eastern populations, and factors such as high rates of smoking and dust exposure in certain areas contribute to a substantial burden of chronic airway diseases.^[7,29]^ Previously, clinicians and researchers in Arabic-speaking countries used non-validated translations or generic respiratory questionnaires to assess cough-related quality of life. The availability of the LCQ-A fills this gap by providing a reliable, culturally relevant tool specifically designed for cough assessment. Furthermore, the consistency of our results with those from international studies suggests that data collected via the LCQ-A can be meaningfully compared with findings from other countries. To our knowledge, this is the first study to validate the LCQ in Arabic, expanding its availability to a large global population. Therefore, adopting the LCQ-A in routine care could enhance the recognition of the broad effects of cough on patient well-being and inform more holistic management approaches in populations such as those in Saudi Arabia and its neighboring countries.

## 5. Conclusion

In conclusion, our study establishes the LCQ-A as a reliable and valid instrument for assessing chronic cough in Arabic speakers. The use of this instrument is expected to facilitate more accurate clinical assessment and research on the impact of cough in this population.

## Acknowledgments

The authors extend their appreciation to Umm Al-Qura University, Saudi Arabia, for funding this research work through grant number 26UQU4340488GSSR03.

## Author contributions

**Conceptualization:** Saeed M. Alghamdi, Maryam M. Almulhem, Surinder S. Birring.

**Data curation:** Saeed M. Alghamdi, Ahmed H. Alasimi, Saja A. Almulhim, Abdulelah M. Aldhahir, Jaber S. Alqahtani, Surinder S. Birring.

**Formal analysis:** Saeed M. Alghamdi, Ahmed H. Alasimi, Maryam M. Almulhem, Rayan A. Siraj, Surinder S. Birring.

**Investigation:** Saeed M. Alghamdi, Maryam M. Almulhem, Saja A. Almulhim, Abdulelah M. Aldhahir, Jaber S. Alqahtani, Rayan A. Siraj, Surinder S. Birring.

**Methodology:** Saeed M. Alghamdi, Ahmed H. Alasimi, Maryam M. Almulhem, Saja A. Almulhim, Jaber S. Alqahtani, Rayan A. Siraj, Surinder S. Birring.

**Project administration:** Saeed M. Alghamdi, Abdulelah M. Aldhahir, Jaber S. Alqahtani, Rayan A. Siraj, Surinder S. Birring.

**Resources:** Saeed M. Alghamdi, Ahmed H. Alasimi, Maryam M. Almulhem, Saja A. Almulhim, Abdulelah M. Aldhahir, Rayan A. Siraj, Surinder S. Birring.

**Software:** Saeed M. Alghamdi, Ahmed H. Alasimi, Saja A. Almulhim.

**Supervision:** Saeed M. Alghamdi, Surinder S. Birring.

**Validation:** Saeed M. Alghamdi, Ahmed H. Alasimi, Maryam M. Almulhem, Saja A. Almulhim, Abdulelah M. Aldhahir, Jaber S. Alqahtani, Rayan A. Siraj, Surinder S. Birring.

**Writing – original draft:** Saeed M. Alghamdi, Ahmed H. Alasimi, Maryam M. Almulhem, Saja A. Almulhim, Abdulelah M. Aldhahir, Jaber S. Alqahtani, Rayan A. Siraj, Surinder S. Birring.

**Writing – review & editing:** Saeed M. Alghamdi, Ahmed H. Alasimi, Maryam M. Almulhem, Saja A. Almulhim, Abdulelah M. Aldhahir, Jaber S. Alqahtani, Rayan A. Siraj, Surinder S. Birring.
